# Effects of dietary vitamin D_3_ supplementation on growth performance, blood vitamin D status, and antioxidant capacity in weaning pigs

**DOI:** 10.5713/ab.25.0525

**Published:** 2025-10-22

**Authors:** Chan Ho Kwon, Eva S. Safaie, Jannell A. Torres, Zhaohui Yang, Xi Chen, Young Dal Jang

**Affiliations:** 1 Department of Animal and Dairy Science, University of Georgia, Athens, GA, USA; 2 Nutribins LLC, Covina, CA, USA

**Keywords:** Antioxidant Status, Growth Performance, Oxidative Stress, Vitamin D_3_, Weaning Pig

## Abstract

**Objective:**

This study evaluated the effects of dietary vitamin D_3_ (VD_3_) supplementation on growth performance, blood vitamin D, and antioxidant status in weaning pigs.

**Methods:**

Forty newly weaned piglets (6.02±1.17 kg) were assigned to two treatments with five replicates over a 28-d period. Treatments were 1) NRC-VD_3_: NRC recommended levels (220 IU/kg in Phase 1 [d 0–14 postweaning] and 200 IU/kg in Phase 2 [d 14–28 postweaning]), and 2) High-VD_3_: a high level of VD_3_ (2,000 IU/kg in Phase 1 and 2). Body weight, average daily gain, average daily feed intake, and gain-to-feed ratio were measured weekly. Blood samples were collected at d 14 and 28 postweaning for the analyses of plasma 25-hydroxycholecalciferol (25-OHD_3_), total antioxidant capacity (T-AOC), superoxide dismutase (SOD) activity, and malondialdehyde (MDA) levels. Pearson correlation coefficients between plasma 25-OHD_3_ and SOD, MDA, or T-AOC were determined.

**Results:**

Growth performance did not differ in overall nursery period although feed intake was lower in the High-VD_3_ group than the NRC-VD_3_ group in d 14–28 postweaning (p<0.05). Pigs fed High-VD_3_ diets showed greater plasma 25-OHD_3_ at d 14 and 28 postweaning (p<0.05), tended to have reduced plasma MDA (p = 0.06), and increased plasma SOD activity (p = 0.10) at d 14 postweaning compared with those fed NRC-VD_3_ diets with no effect in plasma T-AOC. At d 14 postweaning, plasma 25-OHD_3_ was positively correlated with plasma SOD activity (r = 0.532; p<0.05) and tended to be negatively correlated with plasma MDA levels (r = −0.491; p = 0.06).

**Conclusion:**

High VD_3_ supplementation at 2,000 IU/kg did not enhance growth performance, while improving plasma vitamin D and antioxidant status in weaning pigs compared to NRC-level supplementation. Therefore, supplementing weaning pigs with higher-than-recommended levels of VD_3_ could be beneficial to enhance their antioxidant status and overall health.

## INTRODUCTION

Vitamin D is essential for maintaining calcium and phosphorus balance and bone health, as well as supporting oxidative defense and immune function, particularly in weaning pigs [1–4]. Vitamin D exhibits antioxidant effects partly by inducing the synthesis of metallothionein, a protein known for its ability to scavenge reactive oxygen species and thereby reduce oxidative stress [5,6]. It plays a critical role in regulating the immune system and inflammatory responses in pigs by reducing pro-inflammatory cytokine levels and supporting intestinal barrier integrity during immune challenges [7].

Piglets are born with naturally low plasma vitamin D levels, and the confined housing conditions commonly used in swine production limit their sunlight exposure, thereby restricting the production of vitamin D in the skin due to reduced exposure to ultraviolet sunlight [8]. In addition, pigs are weaned with a low level of vitamin D in the blood due to insufficient vitamin D levels in milk [9,10]. This emphasizes the importance of providing enough dietary vitamin D to meet the requirements of young pigs. In the current swine industry, dietary vitamin D_3_ (VD_3_) supplementation for nursery pigs typically ranges from 1,600–2,600 IU/kg, which is 8–12 times greater than the NRC [11] recommended levels [12], despite a lack of scientific evidence supporting benefits to growth and health at these high levels. Several studies reported that VD_3_ supplementation positively influenced immune responses and antioxidant status in pigs [7,13], while no effect was observed in growth performance [9,14]. However, previous studies have used lower levels of VD_3_ (e.g., 800 IU) [15] or its metabolites such as 25-hydroxycholecalciferol (25-OHD_3_) [4,16] with varying supplementation methods, including oral administration, drinking water and intramuscular injection [2,10]. Thus, there is still limited information regarding the beneficial effects of high dietary VD_3_ supplementation beyond NRC requirement levels on growth performance, plasma vitamin D, and antioxidant status in weaning pigs. Therefore, this study aims to evaluate the effects of dietary VD_3_ supplementation at two levels, NRC recommendation (200–220 IU/kg) and 2,000 IU/kg on growth performance, blood vitamin D status, and antioxidant parameters in weaning pigs.

## MATERIALS AND METHODS

### Animal care

This experiment and sample collections were carried out in an environmentally controlled room at the University of Georgia Large Animal Research Unit.

### Animals, experimental design, and housing

A total of 40 newly weaned pigs (Camborough×PIC337; 6.02±1.17 kg initial body weight; weaned at 17.2±0.95 d of age) were assigned to 2 treatments in 5 replicates with 4 pigs (2 barrows and 2 gilts) per pen based on body weight, breed, and sex in the randomized complete block design. Treatments were: 1) NRC-VD_3_: VD_3_ supplementation at NRC [11] recommended levels - 220 IU/kg in Phase 1 (d 0–14 postweaning) and 200 IU/kg in Phase 2 (d 14–28 postweaning), added to basal diets without supplemental VD_3_, and 2) High-VD_3_: 2,000 IU/kg of VD_3_ supplementation added to the same basal diets. All pigs were housed in nursery pens (1.0×2.0 m^2^) with woven-wire flooring and had ad libitum access to water and feed in an environmentally controlled nursery facility. No creep feed was provided during the suckling period.

### Experimental diets

All pigs were fed corn-soybean meal-based diets in mash form that were formulated to meet or exceed nutrient requirement estimates of NRC [11] for 7–11 kg (Phase 1) and 11–25 kg (Phase 2) of pigs (Table 1). To minimize differences in non-treatment components of the diets, a basal diet was first mixed with a VD_3_-free vitamin premix, divided into two fractions, and each fraction was mixed with respective treatment levels of VD_3_ (NRC recommended level or 2,000 IU/kg) by replacing corn starch.

### Data and sample collection

The pigs were individually weighed at d 0, 7, 14, 21, and 28 postweaning. Pen-based feed disappearance was measured when the pigs were weighed and average daily gain (ADG), average daily feed intake (ADFI), and gain-to-feed (G:F) ratio were calculated.

On d 0, 14, and 28 postweaning, blood samples (10 mL) were collected from eight pigs per treatment (2 pigs per pen from first 4 replicates) selected based on average body weight in each pen via jugular venipuncture in disposable vacutainer tubes containing the anticoagulant K_3_ EDTA (Becton Dickinson). Plasma sample was obtained by centrifugation at 2,500×g for 30 min at 4°C and stored at −80°C until analysis.

### Chemical analysis

Plasma samples were analyzed for 25-OHD_3_ using liquid chromatography-mass spectrometry at Heartland Assays and antioxidant parameters including superoxide dismutase (SOD; Catalog No. 706002; detection limit - 0.005–0.05 U/mL) activity, total antioxidant capacity (T-AOC; Catalog No. 709001; detection limit - 0.000–0.495 mM trolox equivalents), and malondialdehyde (MDA; Catalog No. 10009055; detection limit - 0.625–50 μM) levels using colorimetric kits (Cayman Chemical Company) and a spectrophotometer (Multiskan Skyhigh; Thermo Fisher Scientific).

### Statistical analysis

All data obtained in the current study were analyzed in accordance with a randomized complete block design using the PROC Mixed procedure of SAS (ver. 9.4; SAS Institute). Pen was used as the experimental unit for the analysis of growth performance data. An individual pig was used as an experimental unit for blood analyses. No pigs were excluded from the data. The models included the treatment as a fixed effect and the replicate as a random effect for growth performance and the replicate within pen and pen as random effects for blood parameters. Pearson correlation coefficients between plasma 25-OHD_3_ concentrations and plasma SOD, MDA, or T-AOC at d 14 and 28 postweaning were determined using PROC CORR of SAS with individual values. The least square means were separated using the PDIFF option of SAS. Statistical differences were established at p<0.05 and tendencies were established at 0.05≤p<0.10.

## RESULTS

There were no significant differences between dietary treatments in body weight, ADG, and G:F during the entire experimental period (p>0.10; Table 2). However, pigs in the High-VD_3_ treatment had significantly lower ADFI (p<0.05) than those in the NRC-VD_3_ treatment during d 14 to 28 postweaning, although there were no differences in the other phase and overall period (p>0.10).

The pigs in the High-VD_3_ treatment had greater plasma 25-OHD_3_ concentrations than those in the NRC-VD_3_ treatment at d 14 and 28 postweaning (p<0.05; Table 3). There were no significant differences in plasma T-AOC between dietary treatments (p>0.10; Table 4). Pigs in the High-VD_3_ treatment tended to have greater plasma SOD activity (p = 0.10) than those in the NRC-VD_3_ treatment at d 14 postweaning, while showing lower plasma MDA levels with a tendency (p = 0.06) compared to those in the NRC-VD_3_ treatment. At d 28 postweaning, there were no significant differences in plasma SOD activity or MDA levels between two treatments (p>0.10).

Plasma 25-OHD_3_ concentrations at d 14 postweaning were positively correlated with plasma SOD activity at d 14 postweaning (p<0.05) and showed a tendency for a positive correlation at d 28 postweaning (p = 0.07). Plasma MDA levels at d 14 postweaning showed a tendency for a negative correlation with plasma 25-OHD_3_ concentrations at d 14 postweaning (p = 0.06; Table 5).

## DISCUSSION

VD_3_ has gained growing attention as a nutritional strategy, not only for its established role in Ca and P homeostasis but also for its emerging functions in supporting immune responses and antioxidant defenses [3,15,17]. Vitamin D exerts antioxidant effects by inducing metallothionein synthesis to scavenge reactive oxygen species [5,6], while also regulating immune and inflammatory responses in pigs by reducing pro-inflammatory cytokines and supporting intestinal barrier integrity [7]. Although the NRC [11] recommends 200–220 IU/kg of dietary VD_3_ for nursery pigs, commercial feeding practices often exceed this level, with reported supplementation rates ranging from 1,600 to 2,600 IU/kg [12]. This discrepancy may indicate potential benefits of higher VD_3_ levels under practical conditions; however, scientific evidence validating such practices remains limited. Therefore, the objective of this study was to evaluate the potential benefits of supplementing 2,000 IU/kg of VD_3_ on growth performance, vitamin D status, and antioxidant parameters in weaning pigs, in comparison with the NRC-recommended levels.

In the current study, pigs fed diets supplemented with 2,000 IU/kg of VD_3_ had no difference in body weight, ADG, or G:F throughout the nursery period compared to those fed diets with the NRC-recommended VD_3_ level. This agrees with previous findings [9,14] suggesting that dietary VD_3_ supplementation does not significantly affect growth performance in nursery pigs. However, pigs fed diets with the NRC-recommended VD_3_ level had a greater feed intake in the late nursery period compared to those fed diets with 2,000 IU/kg of VD_3_, although there was no difference in overall feed intake. The underlying cause of this increase is unclear. In the current study, however, pigs fed diets with the NRC-recommended VD_3_ level had relatively lower antioxidant status, as evidenced by greater plasma MDA levels and lower plasma SOD activity observed at d 14 postweaning compared to those fed diets supplemented with 2,000 IU/kg of VD_3_. Hao et al [18] reported that oxidative stress impairs energy homeostasis by increasing the demand for metabolic resources required for cellular repair and antioxidant defense. Thus, it is possible that pigs experiencing elevated oxidative stress potentially due to low VD_3_ intake may divert energy from growth toward maintaining physiological balance, which possibly results in a greater nutritional need. This pattern was evident in the NRC-VD_3_ group, where elevated ADFI, likely reflecting greater metabolic demands, did not result in enhanced growth performance. Instead, both growth rate and feed efficiency in the late nursery period were numerically lower when pigs were fed diets with the NRC-recommended VD_3_ level, suggesting potentially inefficient nutrient utilization in those pigs.

In the current study, plasma 25-OHD_3_ levels clearly increased in pigs fed diets with 2,000 IU/kg of VD_3_ compared to those fed diets with the NRC-recommended VD_3_ level on both d 14 and 28 postweaning. Since 25-OHD_3_ is the main circulating form of vitamin D in the blood, it is widely used as a marker for blood vitamin D status [19]. Our findings align with previous studies that reported greater VD_3_ intake increases blood 25-OHD_3_ levels in weaning pigs [2,9,20]. Although we only measured blood 25-OHD_3_ levels, Burild et al [20] reported that increasing dietary VD_3_ levels enhanced tissue 25-OHD_3_ levels, suggesting that high dietary VD_3_ intake can also improve vitamin D retention throughout the body.

Although previous studies have consistently shown that supplementation of vitamin D using 25-OHD_3_ resulted in dose-dependent increases in circulating blood 25-OHD_3_ concentrations in pigs [16,21,22], the current study found that increasing dietary VD_3_ by tenfold increased plasma 25-OHD_3_ concentrations by only about 40%. This result agrees with Burild et al [20] reporting that pigs fed diets with VD_3_ from 200 to 2,000 IU/kg had about 2.5 times less increase in plasma 25-OHD_3_ concentrations compared to those fed diets with 25-OHD_3_. Thus, this result indicates the lower efficiency of VD_3_ in elevating circulating 25-OHD_3_ compared to its hydroxylated form (25-OHD_3_), likely due to differences in intestinal absorption, hepatic conversion, and systemic utilization. The VD_3_, being highly lipophilic, relies on micelle formation and lymphatic transport and is more prone to sequestration in adipose tissues, whereas 25-OHD_3_, with greater hydrophilicity, is absorbed more efficiently via both portal and lymphatic routes and enters circulation more directly [20,23–25].

In the current study, 2,000 IU/kg of dietary VD_3_ supplementation tended to reduce plasma MDA concentrations at d 14 postweaning, suggesting that high dietary VD_3_ may help alleviate oxidative stress in weaning pigs. Similarly, previous studies using the hydroxylated form of VD_3_ (25-OHD_3_) have demonstrated reductions in plasma MDA concentrations in weaning pigs, supporting the role of improving vitamin D status in mitigating oxidative stress [16,26]. One proposed mechanism involves the active form of vitamin D, 1,25-dihydroxycholecalciferol, enhancing the expression of metallothionein, a protein that scavenges reactive oxygen species and supports redox balance [5,6,27]. In addition, a tendency toward increased plasma SOD activity in pigs fed diets supplemented with 2,000 IU/kg of VD_3_ at d 14 postweaning indicates a potential stimulatory effect of VD_3_ on enzymatic antioxidant defenses. This observation aligns with previous findings where 25-OHD_3_ supplementation significantly increased serum SOD activity in weaning pigs [26]. Therefore, the reduction in plasma MDA, along with increased plasma SOD activity, indicates that high VD_3_ supplementation at 2,000 IU/kg can result in improved overall pig health by reducing oxidative stress and improving antioxidant capacity.

On the other hand, T-AOC activity was not significantly affected by high VD_3_ supplementation in the current study. This result is consistent with a recent study in broilers, where dietary supplementation with 4,000 IU/kg of VD_3_ did not affect serum T-AOC, SOD activity, and MDA levels compared to the control group [28]. Although species differences may exist, these findings suggest that high VD_3_ supplementation does not always enhance systemic antioxidant enzyme activity. Kwon et al [16] also reported that the effects of dietary vitamin D sources on antioxidant enzyme responses may vary depending on multiple factors such as the animal’s oxidative stress level, health status, diet composition, and environmental conditions. Therefore, further research is needed to demonstrate how VD_3_ supplementation can effectively enhance enzymatic antioxidant defenses in weaning pigs.

Based on the observed increases in plasma 25-OHD_3_ and improvements in antioxidant status following 2,000 IU/kg of VD_3_ supplementation, the current correlation analysis revealed relationships between blood vitamin D status and antioxidant parameters in weaning pigs. At d 14 postweaning, plasma 25-OHD_3_ concentrations were positively correlated with plasma SOD activity and tended to be negatively correlated with plasma MDA levels, suggesting that improved vitamin D status can result in enhanced enzymatic antioxidant defense and potentially reduced oxidative stress in early nursery period. Additionally, a trend for a positive correlation was observed between plasma 25-OHD_3_ concentrations at d 14 postweaning and plasma SOD activity at d 28 postweaning, indicating that early improvements in vitamin D status may have prolonged effects on antioxidant responses. Kwon et al [16] reported that plasma MDA levels reached their minimum when plasma 25-OHD_3_ concentrations exceeded approximately 23.7 ng/mL by d 28 post-weaning based on the broken-line analysis. In the current study, however, plasma 25-OHD_3_ levels did not reach this threshold with VD_3_ supplementation at 2,000 IU/kg. This result indicates that increasing blood vitamin D status beyond the level achieved with this dosage may further improve the antioxidant protection system in pigs. In addition, together with our previous work [16], which observed a similar correlation between plasma 25-OHD_3_ and MDA levels, the correlations found in the current study between plasma 25-OHD_3_ and antioxidant markers support that vitamin D requirement in pigs may need to be expressed based on plasma vitamin D status, using 25-OHD_3_ as a biomarker. Additionally, the relative bioefficacy of VD_3_ compared to 25-OHD_3_ may need to be more precisely evaluated.

## CONCLUSION

Dietary supplementation with 2,000 IU/kg of VD_3_ in weaning pigs improved vitamin D status, as evidenced by elevated plasma 25-OHD_3_ concentrations, without affecting growth performance compared to NRC [11] recommended levels. High VD_3_ supplementation at 2,000 IU/kg also reduced oxidative stress and enhanced antioxidant enzyme activity in the early nursery period. In addition, plasma vitamin D status was positively correlated with antioxidant enzyme activity and negatively correlated with an oxidative stress marker. These findings support higher-than-recommended levels of VD_3_ may be a beneficial nutritional strategy to improve antioxidant status and overall health in weaning pigs. Further research is needed to refine dosing strategies and explore the long-term effects of improved vitamin D status on immune function, gut health, and performance outcomes.

## Figures and Tables

**Table 1:** Diet formulation and calculated chemical composition (as-fed basis)

Ingredients (%)	Phase 1 d 0–14 postweaning	Phase 2 d 14–28 postweaning
Corn	38.49	50.83
Soybean meal (48% crude protein)	24.00	28.00
Whey, dried	10.00	10.00
Oats	2.50	2.50
HP3001)	3.00	3.00
Lactose	10.00	0.00
Fish meal	3.00	0.00
Animal plasma	3.00	0.00
Soybean oil	2.00	1.50
Corn starch	1.00	1.00
L-Lysine·HCl	0.21	0.29
DL-Methionine	0.18	0.17
L-Threonine	0.15	0.15
Dicalcium phosphate	0.68	0.74
Limestone	0.95	0.98
Salt	0.50	0.50
Trace mineral mix2)	0.15	0.15
Vitamin mix (vitamin D free)3)	0.20	0.20
Total	100.00	100.00
Calculated chemical composition		
Metabolizable energy (kcal/kg)	3,415	3,360
Crude protein (%)	22.49	21.22
SID lysine (%)	1.35	1.23
SID methionine+cysteine (%)	0.82	0.74
Total Ca (%)	0.80	0.70
Total P (%)	0.63	0.57
STTD P (%)	0.40	0.33

1) Hamlet Protein, Findlay, OH.

2) The trace mineral premix supplied the following per kilogram of diet: 33 mg of Mn as manganous oxide, 110 mg of Fe as ferrous sulfate, 110 mg of Zn as zinc sulfate, 16.5 mg of Cu as copper sulfate, 0.3 mg of I as Ca iodate, 0.3 mg of Se as sodium selenite.

3) The vitamin premix supplied the following per kilogram of diet: 11,000 IU of vitamin A, 99 IU of vitamin E, 4.4 mg of vitamin K, 55 μg of vitamin B_12_, 9.9 mg of riboflavin, 31.9 mg of pantothenic acid, 55 mg of niacin, 0.9 mg of folic acid, 3.9 mg of vitamin B_6_, 3.1 mg of thiamin, and 0.3 mg of biotin, 600 mg of choline chloride. Vitamin D_3_ was added to basal diets at the assigned level for each treatment.

SID, standardized ileal digestible; STTD, standardized total tract digestible.

**Table 2:** Postweaning growth performance of pigs fed diets supplemented with NRC recommended or high level of Vitamin D_3_ (VD_3_)

Item	Treatment1)		SEM	p-value

	NRC-VD_3_	High-VD_3_		
Body weight (kg)				
d 0	6.02	6.00	0.55	0.41
d 7	6.87	6.82	0.59	0.82
d 14	9.71	9.42	0.69	0.34
d 21	12.14	11.98	0.86	0.76
d 28	16.22	16.30	1.10	0.85
Average daily gain (kg/d)				
d 0–7	0.122	0.118	0.021	0.86
d 7–14	0.405	0.371	0.026	0.12
d 14–21	0.348	0.365	0.033	0.69
d 21–28	0.583	0.618	0.062	0.58
d 0–14 (Phase 1)	0.264	0.244	0.021	0.39
d 14–28 (Phase 2)	0.465	0.492	0.037	0.26
d 0–28 (overall)	0.364	0.368	0.029	0.88
Average daily feed intake (kg/d)				
d 0–7	0.216	0.219	0.023	0.92
d 7–14	0.594	0.546	0.042	0.26
d 14–21	0.676	0.635	0.031	0.14
d 21–28	0.918	0.906	0.053	0.46
d 0–14 (Phase 1)	0.405	0.382	0.032	0.46
d 14–28 (Phase 2)	0.797	0.770	0.044	0.01
d 0–28 (overall)	0.601	0.576	0.035	0.16
G:F				
d 0–7	0.561	0.529	0.056	0.66
d 7–14	0.683	0.688	0.032	0.91
d 14–21	0.514	0.571	0.043	0.32
d 21–28	0.631	0.682	0.054	0.48
d 0–14 (Phase 1)	0.650	0.644	0.026	0.68
d 14–28 (Phase 2)	0.583	0.638	0.022	0.11
d 0–28 (overall)	0.606	0.639	0.023	0.21

n = 5 replicate pens per treatment.

1) Treatments were: 1) NRC-VD_3_: 220 IU/kg (Phase 1) and 200 IU/kg (Phase 2) of VD_3_ supplementation based on NRC [11] and 2) high-VD_3_: 2,000 IU/kg of VD_3_ supplementation.

SEM, standard error of the mean; G:F, gain-to-feed.

**Table 3:** Postweaning plasma 25-hydroxycholecalciferol concentrations (ng/mL) in pigs fed diets supplemented with NRC recommended or high level of Vitamin D_3_ (VD_3_)

Item	Treatment1)		SEM	p-value

	NRC-VD_3_	High-VD_3_		
d 14	14.80	20.53	1.165	0.01
d 28	15.66	19.29	0.944	0.03

n = 8 replicate pigs per treatment.

1) Treatments were: 1) NRC-VD_3_: 220 IU/kg (Phase 1) and 200 IU/kg (Phase 2) of VD_3_ supplementation based on NRC [11] and 2) high-VD_3_: 2,000 IU/kg of VD_3_ supplementation.

SEM, standard error of the means.

**Table 4:** Postweaning plasma antioxidant status of pigs fed diets supplemented with NRC recommended or high level of Vitamin D_3_ (VD_3_)

Item	Treatment1)		SEM	p-value

	NRC-VD_3_	High-VD_3_		
Superoxide dismutase (U/mL)				
d 14 postweaning	4.15	5.38	0.459	0.10
d 28 postweaning	5.38	5.55	1.698	0.79
Malondialdehyde (μM)				
d 14 postweaning	10.14	7.90	1.221	0.06
d 28 postweaning	13.71	12.61	0.794	0.35
Total antioxidant capacity (mM trolox equivalents)				
d 14 postweaning	4.38	4.55	0.310	0.67
d 28 postweaning	5.00	4.91	0.320	0.81

n = 8 replicate pigs per treatment.

1) Treatments were: 1) NRC-VD_3_: 220 IU/kg (Phase 1) and 200 IU/kg (Phase 2) of VD_3_ supplementation based on NRC [11] and 2) high-VD_3_: 2,000 IU/kg of VD_3_ supplementation.

SEM, standard error of the means.

**Table 5:** Pearson correlation coefficients between plasma 25-OHD_3_ concentrations and antioxidant parameters in pigs

Item		SOD		MDA		T-AOC	
		
		d 141)	d 28	d 14	d 28	d 14	d 28
25-OHD_3_	d 14	0.532*	0.486**	−0.491**	0.381	0.158	−0.189
	d 28	0.271	0.004	−0.170	0.188	0.124	−0.052

Significant differences:

* p<0.05,

** p<0.10.

1) Days after weaning.

SOD, superoxide dismutase (U/mL); MDA, malondialdehyde (μM); T-AOC, total antioxidant capacity (mM trolox equivalents); 25-OHD_3_, 25-hydroxycholecalciferol (ng/mL).

## Data Availability

Upon reasonable request, the datasets of this study can be available from the corresponding author.
